# Diet moderates the effect of resting state functional connectivity on cognitive function

**DOI:** 10.1038/s41598-022-20047-4

**Published:** 2022-09-27

**Authors:** Alexandra M. Gaynor, Eleanna Varangis, Suhang Song, Yunglin Gazes, Diala Noofoory, Reshma S. Babukutty, Christian Habeck, Yaakov Stern, Yian Gu

**Affiliations:** 1 Taub Institute for Research on Alzheimer’s Disease and the Aging Brain, Columbia University, New York, NY USA; 2 Cognitive Neuroscience Division, Department of Neurology, Columbia University, New York, NY USA; 3 Gertrude H. Sergievsky Center, Columbia University, New York, NY USA; 4 Department of Psychiatry, Columbia University, New York, NY USA; 5 Department of Epidemiology, Joseph P. Mailman School of Public Health, Columbia University, New York, NY USA

**Keywords:** Cognitive ageing, Cognitive neuroscience

## Abstract

Past research suggests modifiable lifestyle factors impact structural and functional measures of brain health, as well as cognitive performance, but no study to date has tested the effect of diet on resting state functional connectivity (rsFC), and its relationship with cognition. The current study tested whether Mediterranean diet (MeDi) moderates the associations between internetwork rsFC and cognitive function. 201 cognitively intact adults 20–80 years old underwent resting state fMRI to measure rsFC among 10 networks, and completed 12 cognitive tasks assessing perceptual speed, fluid reasoning, episodic memory, and vocabulary. Food frequency questionnaires were used to categorize participants into low, moderate, and high MeDi adherence groups. Multivariable linear regressions were used to test associations between MeDi group, task performance, and internetwork rsFC. MeDi group moderated the relationship between rsFC and fluid reasoning for nine of the 10 functional networks’ connectivity to all others: higher internetwork rsFC predicted lower fluid reasoning performance in the low MeDi adherence group, but not in moderate and high MeDi groups. Results suggest healthy diet may support cognitive ability despite differences in large-scale network connectivity at rest. Further research is warranted to understand how diet impacts neural processes underlying cognitive function over time.

## Introduction

A breadth of epidemiological research has suggested healthy diets are associated with better cognition, reduced risk of Alzheimer’s disease (AD), and healthier brain structural measures^1^. One of the most commonly researched diets in relation to cognitive and brain health is the Mediterranean diet (MeDi), which is characterized by high intake of vegetables, legumes, fruits, cereals, and unsaturated fatty acids, low intake of meat and poultry, and a moderate amount of alcohol. Adherence to MeDi has been associated with lower incidence of AD^2^, decreased risk for mild cognitive impairment (MCI)^3^, and slower cognitive decline in multiple domains^2^. Several longitudinal studies have provided further evidence that MeDi may have a protective effect against cognitive aging^4,5^ and potentially reduces risk of MCI, dementia, and AD^2,3,6^.

Relatively few studies have investigated the relationship between diet and brain imaging biomarkers. Nevertheless, a small number of recent studies have provided some evidence that MeDi adherence is significantly associated with structural measures of brain health. Previous research from our group and others has found that cognitively healthy adults demonstrate a positive association between adherence to MeDi or components of MeDi and brain volume^7,8^, cortical thickness^9^, and structural connectivity^10,11^. Furthermore, there is evidence that MeDi may contribute to brain maintenance by reducing neurodegenerative or neuropathological damages. For example, greater MeDi adherence has been associated with slower decline in gray matter^12^, better preserved structural connectivity^13^, lower white matter hyperintensity burden^14^, reduced odds of clinical vascular events^15^, and lower beta amyloid burden and accumulation over time^16,17^.

Although there is evidence MeDi may impact brain structure and protect against neuropathological damage, no research to date has examined the effects of MeDi on brain activity measured by functional MRI. A small number of short-term diet trial studies have identified localized changes in brain activity in response to intake of specific nutrients, suggesting a role for diet in neurocognitive function. However, research on the effects of individual nutrients is limited by a failure to account for the interactive and cumulative effects of multiple nutrients within a comprehensive dietary pattern^3^. Moreover, most previous studies measured immediate or short-term effects of nutrient/food items on brain activation, and whether such effects can be seen with habitual consumption, or be sustained for a longer time, remains unknown. Finally, no research to date has examined the association between diet and functional connectivity between large-scale brain networks at rest, which may underlie cognitive function^18–21^.

Several studies have shown that internetwork resting state connectivity (rsFC), correlations between regions belonging to different large-scale networks, tends to increase (i.e. networks become more inter-dependent and less segregated) with age^22–25^, and meaningful changes in internetwork rsFC may be evident as early as middle age^26^. These increases in rsFC between networks are thought to reflect decreased specialization of functional networks underlying cognitive abilities^22–24^. Indeed, reduced network segregation has been associated with worse cognitive performance in both cross-sectional^18,25,27^ and longitudinal^23^ studies, consistent with evidence that resting-state topology serves a critical role in the transfer and coordination of task information during cognitive processing^21^. The exact reasons for individual variations in rsFC is unclear, but some modifiable lifestyle factors, such as exercise, have been associated with rsFC in healthy older adults^28^, individuals with MCI^28^, and those at risk for dementia^29^. Thus, it is not unreasonable to hypothesize that MeDi, as another key modifiable lifestyle factor associated with cognition and structural brain measures, may also alter patterns of functional connectivity that underlie cognitive function.

Meanwhile, numerous studies have shown that among people with similar brain pathology, there is significant variation in terms of clinical manifestation, including cognitive performance, suggesting certain subgroups of people may better tolerate age- or illness-related brain changes. Recent work from our group has demonstrated that variability in cognitive performance during aging may depend on organization of functional and structural brain networks^18,22^, and lifestyle factors may moderate the relationship between brain measures and cognitive performance^30^. One study also found that higher blood levels of polyunsaturated fatty acids and lycopene moderated the association between network efficiency and cognitive performance^31^. Although this suggests a potential role for specific dietary components in moderating the relationships between brain measures and cognitive function, it remains unknown how a comprehensive dietary pattern such as MeDi may impact the effect of internetwork connectivity on cognition.

The present study aimed to (1) test the relationship between MeDi and overall internetwork rsFC, and (2) test whether MeDi moderates the association between rsFC and cognitive function in this cohort. We hypothesized that individuals who are more adherent to MeDi would demonstrate (1) lower overall internetwork rsFC, and (2) a weaker association between rsFC and cognitive performance, reflecting a potential protective effect of MeDi against cognitive deficits due to changes in rsFC.

## Methods

### Participants

The present study included adults aged 20–80 years, who completed the baseline visit for the Reference Ability Neural Network (RANN) study (N = 425)^32^. For the current analyses, participants were excluded if they did not have diet questionnaire data (108); of the 317 remaining participants, 22 were excluded due to missing rsFC data. Of the 295 remaining participants with diet and rsFC data, 87 were excluded due to missing structural data, and a further 7 were excluded due to missing in-scanner cognitive tests, resulting in a final sample size of N = 201.

All participants were native English speakers, right-handed, free of MRI contraindications, and read at a fourth-grade level or above. No participants had any psychological or medical conditions that could affect cognitive function, and no older adults met criteria for dementia or MCI. The studies were approved by the Institutional Review Board of the College of Physicians and Surgeons of Columbia University. All participants provided informed consent, and all methods were performed in accordance with the relevant guidelines and regulations of the institution and with the ethical standards of the 1964 Declaration of Helsinki.

### Dietary assessment

Habitual dietary information in the past year was self-reported using a 61-item version of Willett’s semi-quantitative food frequency questionnaire (FFQ)^33^. Frequency of consumption in servings per month was calculated for each of 11 food categories and assigned frequency categories ranging from 0 to 5. For beneficial components (non-refined cereals, potatoes, fruits, vegetables, legumes, fish, olive oil), increasing scores corresponded to increased intake; detrimental components (poultry, red meat, full fat dairy products, alcohol) were reverse-scored such that increasing scores corresponded to decreasing frequency. Total MeDi score was calculated by adding scores in food categories (0–55), with higher score indicating higher adherence. MeDi scores were categorized into three groups (low, moderate, high) based on tertiles of total scores. Intake frequency scores were also summed separately for beneficial and detrimental food groups.

### Cognitive function

Cognitive abilities were measured using 12 in-scanner tasks designed to assess performance on four latent variables, or reference abilities (RAs): perceptual speed, fluid reasoning, episodic memory, and vocabulary^32^. *Perceptual speed (SPEED)* was measured using a digit symbol task, letter comparison task, and pattern comparison task. *Fluid reasoning (FLUID)* was measured using a paper folding task, a matrix reasoning task, and a letter set task. *Episodic memory (MEMORY)* was tested using a logical memory task, a word order task, and a paired associates task. *Vocabulary (VOCAB)* was measured using an antonym task, a synonym task, and a picture naming task. (For more detail on task parameters, please see^32^.

Performance on each task was z-scored relative to the mean and standard deviation in participants of the entire RANN study. Z-scores for tasks within each cognitive domain were then averaged to produce four reference ability z-scores for each participant.

### MRI procedures and rsFC assessment

MR images were acquired on a 3 T Philips Achieva Magnet during two study visits lasting approximately 2-h each. Participants underwent 5 min (n = 62) or 9.5 min (n = 129) of fMRI blood oxygen level-dependent (BOLD) resting state scans. T1-weighted whole brain images were acquired for each subject using magnetization-prepared rapid gradient-echo (MPRAGE) sequence. Diffusion MRI (dMRI) and fluid-attenuated inversion recovery (FLAIR) scans were also acquired. Scanning parameters can be found in Supplementary Table S3.

MRI preprocessing included slice-timing correction and motion correction performed in FSL^34^, frame-wise displacement (FWD) calculation, volume replacement for contaminated volumes (scrubbing), band-pass filtering (0.01–0.08 Hz), and residualization of processed data by regressing out FWD, root mean square difference of BOLD signal, left and right hemisphere white-matter, and lateral-ventricular signals. T1 image segmentation was performed using FreeSurfer software and visually inspected for inaccuracies.

dMRI data were preprocessed through standard preprocessing pipeline including eddy- and motion correction using FSL, and TRACULA^35^ toolbox, distributed as part of Freesurfer (v5.2.0), was used to derive mean fractional anisotropy (FA) for 18 major white matter tracts, and averaged to produce a single FA variable for each participant. White matter hyperintensity burden (WMH) was derived from FLAIR imaging using the Lesion Segmentation Toolbox for Statistical Parametric Mapping, and WMH volumes were log transformed prior to analysis. Mean cortical thickness and total brain volume were obtained using T1-weighted MPRAGE scans, and standard FreeSurfer parcellation schemes were used to calculate mean thickness and volume based on 68 cortical regions of interest^36^.

For connectivity analyses, 264 ROIs defined by Power et al.^37^ were transferred to native space via non-linear registration of each subject’s structural scan to MNI template using ANTS software package. A 10 mm radius spherical mask was generated for each coordinate and intersected with the FreeSurfer gray matter mask in order to derive the gray matter-registered ROI masks for each of the 264 ROIs. An intermodal, intra-subject, rigid-body registration of the fMRI reference image and T1 scan was then performed using FLIRT with 6 degrees of freedom, normalized mutual information as the cost function^38^, in order to transfer ROI masks from T1 space to fMRI space. These transferred ROI masks were used to average all voxels within each mask to obtain a single fMRI time-series for each of the 264 ROIs. Time-series data from each ROI were used to generate correlation matrices among all ROIs (264 × 264 ROIs) and were then z-transformed to generate normalized correlation matrices for each participant. ROIs with centers located within 20 mm of one another were set to zero as per Power et al.^37^. All negative correlation values were excluded from mean correlation computations, and average positive correlations were computed between all networks of interest. Although it is possible to include negative correlations in network analyses, there is considerable ambiguity in interpretation of negative correlations^39,40^. Therefore, only positive correlations in each participant’s correlation matrices were included in current analyses^41^.

ROIs were labeled based on the Power et al. network assignments^37^, with the following 10 networks being selected for analysis: Somatomotor Hand (Hand; 30 ROIs), Visual (Vis; 31 ROIs), Somatomotor Mouth (Mouth; 5 ROIs), Auditory (Aud; 13 ROIs), Default Mode (DMN; 58 ROIs), Salience (Sal; 18 ROIs), Cingulo-Opercular (CO; 14 ROIs), Frontoparietal (FP; 25 ROIs), Dorsal Attention (DAN; 11 ROIs), and Ventral Attention (VAN; 9 ROIs). The primary functional connectivity outcome in the present study was the mean internetwork correlation among all 10 networks (1 value reflecting overall mean connectivity between all ROIs comprising all networks). Exploratory network-based analyses then examined network-based internetwork connectivity, or the mean correlation between ROIs in each network and ROIs in the nine other networks, in order to probe network-specific effects (10 values reflecting mean internetwork connectivity from each of the 10 networks to all others).

### Data analyses

Descriptive statistics were used to present participant characteristics including age, education, gender, race, ethnicity, and IQ scores. Multivariable linear regression models were used to examine the relationship between MeDi and overall internetwork rsFC, and interactions between MeDi groups and overall internetwork rsFC on each of the four RAs. All models were adjusted for age, gender, race/ethnicity, education, estimated premorbid intelligence (NART IQ)^42^, total caloric intake, mean cortical thickness, total brain volume, and percentage of motion artifact removed. Given evidence that functional network connectivity is dependent on underlying structure of white matter tracts^43^, we also adjusted for potential confounding effects of total WMH and mean FA.

In supplementary analyses, we performed post-hoc analyses by using the network-level measures of internetwork connectivity. Significant results were further probed by analyzing connectivity between pairs of individual networks. Additional exploratory analyses were conducted to test whether the moderating effect of MeDi on the association between rsFC and cognition was driven by adherence to beneficial foods or avoidance of detrimental foods. Finally, to account for the potential impact of age on the interaction between MeDi and rsFC on cognition, we conducted sensitivity analyses modeling the three-way interaction of age as a continuous variable, MeDi group, and rsFC on each RA. The low MeDi group was set as the reference group in all models. Primary analyses were also conducted excluding participants with scrub percentage greater than 30 (N = 4); results did not differ from those using the full sample (data not shown).

All analyses were conducted in SPSS (version 27.0). Two-sided *p* < 0.05 indicated significance, except for p values of interaction terms with a significance level of 0.10.

## Results

### Descriptive analyses

The participants were on average 54.10 (SD = 15.98, range 21–80) years old, and had a mean of 16.14 (SD = 2.26, range 12–22) years of education. About 53% were female, and 66.2%, 22.4% and 11.4% of participants identified as non-Hispanic white, non-Hispanic black, and other race/ethnicity, respectively. Mean NART IQ score was 117.28 (SD = 8.10, range 93.60–130.88).

One-way analyses of variance showed there were no significant differences between MeDi groups in mean age (*F*[2,200] = 2.269, *p* = 0.106), years of education (*F*[2,200] = 0.847, *p* = 0.430), or NART IQ (*F*[2,200] = 0.437, *p* = 0.647) (Table 1). There were no significant differences in mean cortical thickness (*F*[2,200] = 1.373, *p* = 0.256), total brain volume (*F*[2,200] = 1.304, *p* = 0.274), WMH (*F*[2,200] = 0.074, *p* = 0.929), DTI FA (*F*[2,200] = 0.909, *p* = 0.405), or total scrub percentage (*F*[2,200] = 0.751, *p* = 0.473). There were no group differences in overall internetwork rsFC (*F*[2,200] = 0.514, *p* = 0.599), or performance on the four RAs: FLUID (*F*[2,200] = 0.355, *p* = 0.702), MEMORY (*F*[2,200] = 0.121, *p* = 0.886), VOCAB (*F*[2,200] = 0.632, *p* = 0.533), SPEED (*F*[2,200] = 0.067, *p* = 0.935). Chi-squared tests showed MeDi groups did not differ by race (*X*^2^ = 0.739, p = 0.946), but females were more likely to have middle and high tertile of MeDi than males (*X*^2^ = 9.938, p = 0.007) (Table 1). Associations between rsFC and demographic and cognitive variables can be found in Supplementary Table S2.

**Table 1:** Participant characteristics by MeDi group.

	Low MeDi	Moderate MeDi	High MeDi	Sig*.* (*p*)
N	66	67	68	
Age (years)	51.11 (16.64)	56.97 (14.48)	54.19 (16.43)	0.106
Education (years)	16.14 (2.33)	16.40 (2.37)	15.89 (2.07)	0.430
% Female	37.9	64.2	57.4	0.007**
% Non-Hispanic white	63.6	65.7	69.1	0.946^a^
% Non-Hispanic black	24.2	23.9	19.1	–
% Other race/ethnicity	12.1	10.4	11.8	–
NART IQ	116.74 (8.52)	118.01 (8.40)	117.08 (7.42)	0.647
Mean cortical thickness	2.58 (0.12)	2.53 (0.10)	2.55 (0.13)	0.256
Total brain volume	1509.00 (51,458.97)	− 7347.06 (44,941.06)	6405.33 (53,462.90)	0.274
WMH	1162.80 (3235.90)	1128.51 (3359.58)	997.38 (4129.21)	0.929
DTI FA	0.443 (0.022)	0.440 (0.020)	0.445 (0.022)	0.405
Scrub %	4.94 (7.68)	6.86 (11.60)	5.96 (7.12)	0.473
Overall rsFC	0.252 (0.044)	0.247 (0.046)	0.245 (0.045)	0.599
FLUID	− 0.032 (0.813)	0.064 (0.868)	0.085 (0.839)	0.702
MEMORY	0.034 (0.683)	− 0.033 (0.859)	− 0.011 (0.800)	0.886
VOCAB	0.231 (0.753)	0.092 (0.899)	0.094 (0.796)	0.533
SPEED	0.034 (0.804)	0.062 (0.736)	0.084 (0.787)	0.935

Mean (SD) reported for continuous outcomes, percentages reported for categorical outcomes.

p-values reflect differences between MeDi groups based on one-way ANOVAs and Pearson’s chi-square tests.

*WMH* white matter hyperintensities, *DTI FA* fractional anisotropy, *Scrub % *percentage of motion artifact removed, *FLUID* fluid reasoning, *MEMORY* episodic memory, *VOCAB* vocabulary, *SPEED* perceptual speed.

**p < 0.01.

^a^p-value reflects chi-square test of all 3 race/ethnicity categories by MeDi group.

### Association between MeDi and overall inter-network rsFC

MeDi was not associated with overall rsFC among all 10 networks (*p* = 0.784), nor was it associated with rsFC between any individual network and all others (*p* > 0.05 for all); however, regression coefficients were negative for 8 of the 10 networks (data not shown), suggesting that higher MeDi adherence seemed to be associated with lower internetwork rsFC.

### Interaction between MeDi and overall rsFC on cognition

**Figure 1:**
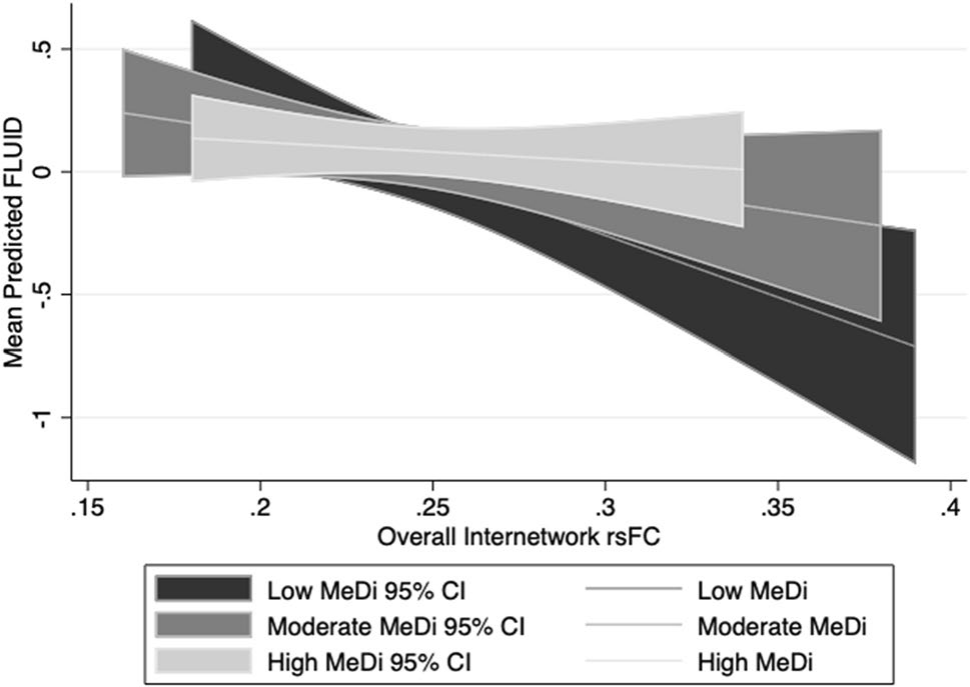
Interaction between effects of MeDi group and overall rsFC on mean predicted fluid reasoning performance. Error bands reflect 95% confidence intervals for mean predicted fluid reasoning for each MeDi group. *FLUID* fluid reasoning performance, *CI* confidence interval.

For FLUID, we found a marginally significant interaction between MeDi group and overall rsFC on performance (*p-interaction* = 0.083): relative to the low MeDi group, the association between overall rsFC and FLUID was weaker in the moderate MeDi group (*p-interaction* = 0.039) and high MeDi group (*p-interaction* = 0.075) (Table 2, Fig. 1). Stratified analyses showed that rsFC had a significant negative effect on FLUID in the low MeDi group (B = − 5.354 [− 8.837, − 1.870], *p* = 0.003), but not in the moderate MeDi group (B = − 0.605 [− 4.521, 3.311], *p* = 0.762) or high MeDi group (B = 0.177 [− 3.681, 4.035], *p* = 0.928) (Table 2). MeDi did not moderate the effect of overall rsFC on VOCAB (*p*-*interaction* = 0.140), but there was a marginally significant difference between the high and low MeDi groups (*p-interaction* = 0.054) in terms of the association between rsFC and VOCAB (Table 2.) MeDi did not moderate the association between overall rsFC and MEMORY or SPEED (Table 2).

**Table 2:** Interaction between MeDi and overall rsFC on each reference ability.

	Overall interaction between MeDi groups and rsFC	Post-hoc analysis: Comparing association of rsFC with reference abilities between moderate (or high) and low MeDi			
		Moderate vs. low MeDi		High vs. low MeDi	
	*p*	B [LL, UL]	*p*	B [LL, UL]	*p*
FLUID	0.083^†^	5.620 [0.286, 10.954]	0.039*	4.779 [− 0.479, 10.037]	0.075^†^
MEMORY	0.587	2.618 [− 2.581, 7.816]	0.324	0.628 [− 4.506, 5.763]	0.810
VOCAB	0.140	2.770 [− 1.149, 6.690]	0.116	3.879 [− 0.059, 7.817]	0.054^†^
SPEED	0.303	0.687 [− 4.115, 5.490]	0.779	− 2.934 [07.888, 2.021	0.246

B = unstandardized regression coefficient for the interaction, with 95% Wald confidence intervals [LL: limit, UL: upper limit].

*FLUID* fluid reasoning, *MEMORY* episodic memory, *VOCAB* vocabulary, *SPEED* perceptual speed.

*p < 0.05; ^†^*p* < 0.10.

### Exploratory analyses

#### Average rsFC for each network

In exploratory analyses of network-based rsFC, we found significant or marginally significant effects of MeDi on the association between rsFC and FLUID for Hand, Visual, Auditory, DMN and DAN networks (Supplementary Table S1). Specifically, for all but the VAN network, rsFC between each network and all others was significantly negatively associated with FLUID in the low MeDi group, but not in moderate or high MeDi groups (Table 3). MeDi was a marginally significant moderator of the relationship between rsFC and VOCAB for several specific networks: compared to the low MeDi group, auditory, fronto-parietal, cingulo-opercular, and dorsal attention networks showed more positive relationships between rsFC to all other networks and VOCAB performance in moderate and high MeDi groups, reflecting a weaker negative effect of rsFC on performance with higher MeDi adherence (Table S1). MeDi significantly moderated effects of rsFC on MEMORY for the Mouth network (*p-interaction* = 0.007), and the Auditory network (*p-interaction* = 0.047), where higher MeDi groups had a stronger association between rsFC and performance, relative to the low MeDi group (Table S1). There were no significant interactions between MeDi and rsFC on SPEED performance for any individual networks’ connectivity to all other networks (Table S1).

**Table 3:** Interactions between MeDi and rsFC on fluid reasoning performance.

Network	Effects of rsFC on FLUID within MeDi groups					
	Low MeDi		Moderate MeDi		High MeDi	
	B [LL, UL]	*p*	B [LL, UL]	*p*	B [LL, UL]	*p*
All	− 5.354 [− 8.837, − 1.870]	0.003**	− 0.605 [− 4.521, 3.311]	0.762	0.177 [− 3.681, 4.035]	0.928
Hand	− 4.355 [− 7.145, − 1.565]	0.002**	1.621 [− 1.758, 5.000]	0.347	− 1.180 [− 4.634, 2.274]	0.503
Vis	− 3.998 [− 6.554, − 1.452]	0.002**	− 0.257 [− 2.881, 2.367]	0.848	0.964 [− 2.555, 4.483]	0.591
Mouth	− 2.507 [− 4.853, − 0.161]	0.036*	1.187 [− 1.312, 3.687]	0.352	− 1.547 [− 4.149, 1.055]	0.244
Aud	− 3.393 [− 6.632, − 0.144]	0.041*	1.227 [− 2.435, 4.889]	0.511	2.444 [− 0.547, 5.435]	0.109
DMN	− 4.858 [− 8.500, − 1.217]	0.009**	− 1.683 [− 5.349, 1.984]	0.368	− 0.249 [− 3.631, 3.133]	0.142
FP	− 3.074 [− 6.000, − 0.148]	0.039*	− 1.942 [− 6.094, 2.209]	0.359	− 0.635 [− 4.569, 3.298]	0.752
VAN	− 0.926 [− 4.107, 2.254]	0.568	− 0.891 [− 4.221, 2.439]	0.600	1.938 [− 0.970, 4.845]	0.191
CO	− 3.142 [− 6.498, 0.215]	0.067^†^	− 0.654 [− 4.118, 2.809]	0.711	0.793 [− 2.723, 4.309]	0.659
DAN	− 3.921 [− 6.659, − 1.184]	0.005**	0.094 [− 2.769, 2.958]	0.949	− 0.661 [− 3.823, 2.502]	0.682
Sal	− 4.299 [− 7.745, − 0.852]	0.015*	− 2.358 [− 6.793, 2.077]	0.297	0.577 [− 3.026, 4.181]	0.754

B = unstandardized regression coefficient, with 95% Wald confidence intervals [LL: limit, UL: upper limit].

*FLUID* fluid reasoning, *All* overall inter-network rsFC, *Vis* Visual, *Aud* Auditory, *DMN* default mode network, *FP* fronto-parietal, *VAN* ventral attention network, *CO* cingulo-opercular, *DAN* dorsal attention network, *Sal* salience.

*p < .05; **p < .01; ***p < .001; †p < 0.10.

#### Pairwise rsFC

Because FLUID was the RA on which we found significant interaction effects between MeDi and overall rsFC, as well as consistent effects for nearly all individual networks, we conducted additional exploratory analyses to further probe whether significant interactions on FLUID were driven by rsFC between any specific individual network pairs. Analyses of rsFC between all pairs of networks showed that MeDi group only significantly moderated the relationship between DMN-FP connectivity and FLUID: compared to the low MeDi group, the effect of DMN-FP connectivity on performance was less negative for the moderate MeDi group (*p-interaction* = 0.023) and the high MeDi group (*p-interaction* = 0.003). Although effects on other network pairings were not significant, all interaction estimates between MeDi and rsFC were consistently positive, i.e., a weaker negative effect of rsFC on FLUID was found in those with higher MeDi compared to those with lower MeDi (data not shown).

#### Beneficial or detrimental food scores

There were no significant interactions between beneficial or detrimental food scores and rsFC on FLUID, with the exception of a marginally significant effect of detrimental food scores on connectivity between the auditory network and all other networks (B = 0.511 [− 0.052, 1.075], *p* = 0.075). Nevertheless, for all networks, the interaction effects between detrimental food scores and rsFC on FLUID seemed to be consistently stronger than the interaction between beneficial food scores and rsFC (Table 4), suggesting avoidance of detrimental food groups may drive the moderating effects of MeDi observed in the primary analyses.

**Table 4:** Interactions between effects of MeDi food type (beneficial, detrimental) and rsFC on fluid reasoning performance.

Network	rsFC * Beneficial		rsFC * Detrimental	
	B [LL, UL]	*p*	B [LL, UL]	*p*
All	0.028 [− 0.427, 0.484]	0.903	0.337 [− 0.224, 0.898]	0.239
Hand	0.025 [− 0.356, 0.407]	0.898	0.257 [− 0.200, 0.714]	0.271
Vis	0.037 [− 0.359, 0.433]	0.855	0.345 [− 0.103, 0.793]	0.132
Mouth	− 0.095 [− 0.453, 0.262]	0.601	0.058 [− 0.390, 0.506]	0.799
Aud	0.072 [− 0.344, 0.488]	0.735	0.511 [− 0.052, 1.075]	0.075^†^
DMN	0.015 [− 0.394, 0.424]	0.944	0.211 [− 0.303, 0.725]	0.421
FP	− 0.022 [− 0.463, 0.419]	0.922	0.205 [− 0.342, 0.752]	0.463
VAN	0.128 [− 0.291, 0.546]	0.549	0.347 [− 0.184, 0.878]	0.200
CO	− 0.023 [− 0.459, 0.413]	0.918	0.311 [− 0.232, 0.855]	0.261
DAN	0.070 [− 0.333, 0.474]	0.732	0.164 [− 0.312, 0.639]	0.500
Sal	0.042 [− 0.397, 0.481]	0.850	0.416 [− 0.172, 1.005]	0.166

B = unstandardized regression coefficient, with 95% Wald confidence intervals [LL: limit, UL: upper limit].

*All* overall inter-network rsFC, *Vis* Visual, *Aud* Auditory, *DMN* default mode network, *FP* fronto-parietal, *VAN* ventral attention network, *CO* cingulo-opercular, *DAN* dorsal attention network, *Sal* salience.

^†^*p* < .10.

#### Effects of demographics on interaction between MeDi and rsFC

For MEMORY, there was a marginally significant interaction among MeDi, rsFC, and age for the hand network (*p-interaction* = 0.079) and the visual network (*p-interaction* = 0.079). More specifically, the reduced association between rsFC and MEMORY with increasing MeDi score was less prominent with increasing age. That is, in older subjects, MeDi can mitigate the negative effect of de-differentiation on RA to a lesser extent than in younger adults. There were no significant three-way interactions between MeDi, rsFC, and age on other three RAs (FLUID, VOCAB, SPEED). There was a marginally significant interaction among MeDi, rsFC, and gender on FLUID for the cingulo-opercular network (*p*-interaction = 0.100) and a significant interaction on FLUID in the auditory network (*p-interaction* = 0.027), both with the interaction between rsFC and MeDi more prominent for females than for males. In contrast, for the mouth network, males showed a more prominent interaction between rsFC and MeDi on FLUID than females (*p*-interaction = 0.047). There were no significant three-way interactions between MeDi, rsFC, and gender on MEMORY, VOCAB, or SPEED.

## Discussion

In the current cross-sectional study, we found that MeDi was not directly associated with rsFC, but could attenuate the negative association between internetwork rsFC and fluid reasoning performance in multiple functional networks, suggesting MeDi has a protective effect on cognitive performance in the face of decreased segregation between large-scale functional networks. To our knowledge, this is the first study to test the relationship among MeDi, functional connectivity, and cognitive performance, and demonstrate that MeDi may benefit cognition by mitigating the negative effect of increased internetwork connectivity on cognitive function.

Of the four reference abilities tested, MeDi had the most robust effect on the relationship between internetwork rsFC and fluid reasoning. This is consistent with past research from our group demonstrating that during fluid reasoning tasks, performance was significantly correlated with connectivity between multiple networks, whereas vocabulary, speed, and memory domains showed relatively fewer correlations with internetwork connectivity^18^. Other researchers have reported similar findings, with fluid reasoning tasks being associated with functional connectivity in attention, decision-making, sensorimotor, visual, and default mode networks^44^. Given that fluid reasoning is thought to be a core aspect of fluid intelligence, and relies on complex integration of multiple higher-order cognitive processes, it is therefore likely that engagement in these tasks recruits multiple large-scale brain networks. Although MeDi did not significantly moderate the effect of overall rsFC on any of the other reference abilities, it is worth noting that there was a marginally significant difference between the effect of overall rsFC on VOCAB in the high MeDi group relative to low MeDi group. However, because vocabulary performance tends to significantly improve with age, these results are less clearly interpretable, as any trends could be driven by effects of age on both vocabulary performance and on rsFC.

Our findings could potentially be interpreted in the context of cognitive reserve (CR), the properties of the brain that allow for better than expected cognitive performance given the degree of injury- or age-related changes to the brain^45^. The present study suggests MeDi may contribute to CR by buffering against potential negative effects of increased internetwork rsFC on cognitive function. Nevertheless, rsFC has previously been considered a biomarker of CR, as connectivity serves as the differentiating factor in the association between structural brain measures and cognition^46^. For instance, recent work from our group identified a resting state connectivity pattern which moderated the relationship between cortical thickness and cognitive performance^46^, and which is significantly correlated with IQ, a common proxy for CR. In the current study, we did not find MeDi to be directly associated with rsFC; however, its role in moderating the effects of rsFC on cognition suggest MeDi may exert CR at a more downstream point, such that it impacts task-related neural mechanisms over and above those predicted by rsFC.

The biological mechanisms by which MeDi contributes to neurocognitive health remain unclear, but past research has suggested components of MeDi may alter cytokine activity and cell apoptotic pathways to reduce inflammation and oxidative stress^47^. Adherence to MeDi has also been positively associated with telomere length, a biomarker of aging that is predictive of neurodegenerative disease^48^. Past research has further suggested MeDi may lower risk of cognitive decline by protecting against multiple chronic diseases that are associated with cognitive impairment, such as cardiovascular disease, hypertension, and diabetes^49^. Results of our exploratory analyses suggest avoidance of detrimental foods, including those known to be associated with increased risk for the above chronic illnesses, may underlie the moderating effect of MeDi on the relationship between rsFC and cognition. Therefore, it is plausible that MeDi has an indirect effect on neurocognitive health, whereby it protects against other diseases that may have detrimental effects on neural and cognitive function. However, the majority of existing research has focused only on the effects of beneficial nutrients on neurocognitive outcomes, and further research is needed to understand the respective roles of beneficial and detrimental foods on neural and cognitive function. In addition to understanding the impact of type and amount of food ingested, it may be beneficial to also consider the effect of eating schedules on cognitive and brain health. Indeed, there is a growing body of research suggesting intermittent fasting and caloric restriction may have benefits for neurocognitive health, including possibly protecting against neuropathological damage by reducing oxidative stress and supporting synaptic plasticity^50–52^. As such, future research would further benefit from not only examining the types of foods, but also the regularity of food intake, on neural and cognitive function.

To our knowledge, this is the first study to examine the effects of dietary patterns on rsFC, and to evaluate the moderating effect of MeDi on the relationship between rsFC and cognitive performance. Furthermore, by controlling for the potential confounding effects of structural brain measures and participant characteristics, we were able to demonstrate the direct role of diet, irrespective of other factors known to impact rsFC and cognitive function. The current study is further strengthened by the inclusion of both younger and older adults, as opposed to much of the existing literature exploring the role of MeDi and cognition in only middle-aged or older adults. Although our primary analyses controlled for potential effects of age, exploratory analyses showed that increased age reduced the protective effect of MeDi only in the memory domain, and in two rsFC networks, suggesting the effects of MeDi on the relationship between other rsFC networks and cognitive functions may be present across the adult lifespan.

The limited past research on the effects of diet on brain biomarkers has largely focused on individual nutrients, and has produced inconsistent results, likely because typical diets contain combinations of nutrients that are highly correlated within food items, and which may interact in their effects on neurological and cognitive outcomes^3,53^. The current study, in contrast, is strengthened by the use of a MeDi score that captures intake of multiple food types, which reflects a more comprehensive measure of overall dietary patterns that may be associated with neurocognitive health. The study is further strengthened by the analyses of beneficial compared to detrimental food groups that contribute to the MeDi score, the results of which suggest avoidance of detrimental foods may play a stronger role than consumption of beneficial foods in supporting neurocognitive function. However, it should be noted that even within detrimental and beneficial food groups, there are multiple ways to reach a high MeDi score depending on specific foods consumed and, as such, future research should explore which combinations of food types best predict differences in the association between rsFC and cognitive function. Moreover, past research has typically focused on effects of acute or short-term consumption of dietary nutrients, which do not address the potential cumulative effects of prolonged intake of dietary factors. The current study is strengthened by the use of habitual intake of food types, which significantly contributes to our understanding of how long-term dietary patterns impact neurocognitive function.

One limitation of the current study is the use of a cross-sectional design, which did not allow us to assess the longitudinal relationship between diet, connectivity, and cognition. As such, we are unable to draw conclusions about the benefits of MeDi over time, particularly in the face of age-related changes in neural and cognitive function. Although results of our sensitivity analyses suggested the protective effects MeDi did not differ as a function of age, further longitudinal research is needed to thoroughly understand the effects of MeDi on neurocognitive function throughout the lifespan. Furthermore, because the current study is the first to test the relationships between rsFC, cognition, and diet, and has a relatively limited sample size to power complex interaction models, we set a somewhat liberal significance threshold for interpreting interaction effects. Although a lower significance threshold could increase the chance of Type I errors, the associated effect sizes and confidence intervals demonstrate reasonably robust interaction effects, and warrant replication using larger sample sizes.

We used in-scanner cognitive tasks to assess each of the RAs, as they were administered in the same session as rsFC data were collected, and could thus provide an optimal association between rsFC and cognitive performance. Nevertheless, a measure of RA that incorporates both in-scanner tasks and out-of-scanner neuropsychological tests may provide a more comprehensive measure of RA, and future research would benefit from evaluating whether the relationships between cognitive abilities, rsFC, and diet differ with the types of cognitive tests used.

Another potential limitation of the current study is the use of an externally derived network parcellation scheme, rather than basing network assignments on study participants’ network structures. However, given the wide age range of participants in the current sample, the use of an external parcellation scheme was optimal to avoid age-related biases in network assignment. Moreover, previous research has successfully utilized the current parcellation scheme to test relationships between functional connectivity and cognition^18,22,54^, and the use of an externally-derived taxonomy allows for more accurate reproducibility of our results in other samples. Nevertheless, future research may benefit from deriving functional networks based on study participants and investigate whether MeDi moderates the strength of these networks’ association with cognitive outcomes, to understand the role of network taxonomy more fully in the relationship between rsFC, diet, and cognitive function.

Lastly, we focused on internetwork rsFC, rather than examining rsFC within specific networks, based on existing research from our group and others’ suggesting connectivity between networks may be more predictive of changes in cognitive function, whereas the effects of intranetwork connectivity on performance are more variable, with the direction of associations with cognition depending on which specific networks and cognitive domains are assessed^18,41,55–57^. Further research examining intranetwork rsFC is warranted to understand the potential effects of lifestyle factors on specific networks and their effects on distinct cognitive functions.

## Conclusions

Results of the current study suggest that MeDi may support cognitive function despite changes in brain connectivity, as evidenced by an attenuated negative effect of internetwork connectivity on cognitive function in those with higher MeDi adherence. Future research is warranted to understand how MeDi impacts structural and functional brain biomarkers and cognitive function over time, and to understand which precise patterns of nutrient intake may provide optimal protection against neurocognitive decline.

## Supplementary Information

Supplementary Information.

## Data Availability

The data generated during and/or analyzed during the current study are available from the corresponding author on reasonable request.
