# Supplementary material for: Diet moderates the effect of resting state functional connectivity on cognitive function
Source: Sci Rep. 2022 Sep 27;12:16080. doi: 10.1038/s41598-022-20047-4 (PMC9515193; doi:10.1038/s41598-022-20047-4)
Supplement: Supplementary file 1 — Supplementary Information. [file 41598_2022_20047_MOESM1_ESM.docx]

Supplementary Table S1. Interactions between effects of MeDi group and rsFC between each network and all others.

|  |  | Parameter estimates for rsFC*MeDi group comparisons | | | |
| --- | --- | --- | --- | --- | --- |
|  | rsFC * MeDi | Moderate vs. Low MeDi | | High vs. Low MeDi | |
|  | *p* | B [LL, UL] | *p* | B [LL, UL] | *p* |
| FLUID |  |  |  |  |  |
| All | .083† | 5.620 [0.286. 10.954] | .039* | 4.779 [-0.479, 10.037] | .075† |
| Hand | .026* | 6.139 [1.682, 10.596] | .007** | 2.835 [-1.582, 7.253] | .208 |
| Vis | .050† | 4.361 [0.607, 8.114] | .023* | 4.043 [-0.207, 8.355] | .066† |
| Mouth | .111 | 3.395 [-0.192, 6.983] | .064† | 0.401 [-3.364, 4.166] | .834 |
| Aud | .060† | 4.749 [-0.159, 9.656] | .058† | 5.260 [0.621, 9.898] | .026* |
| DMN | .087† | 5.265 [-0.079, 10.609] | .053† | 5.267 [0.125, 10.410] | .045* |
| FP | .604 | 1.647 [-3.470, 6.764] | .528 | 2.411 [-2.508, 7.329] | .337 |
| VAN | .362 | 1.747 [-2.852, 6.347] | .457 | 3.160 [-1.185, 7.505] | .154 |
| CO | .337 | 3.449 [-1.460, 8.458] | .167 | 2.980 [-1.959, 7.919] | .237 |
| DAN | .061† | 4.917 [0.814, 9.019] | .019* | 3.275 [-1.143, 7.692] | .146 |
| Sal | .360 | 2.334 [-3.367, 8.036] | .422 | 3.792 [-1.417, 9.001] | .154 |
| MEMORY |  |  |  |  |  |
| All | .587 | 2.618 [-2.581, 7.816] | .324 | 0.628 [-4.506, 5.763] | .810 |
| Hand | .237 | 3.570 [-0.796, 7.936] | .109 | 0.563 [-3.740, 4.867] | .797 |
| Vis | .509 | 1.383 [-2.271, 5.036] | .458 | -0.979 [-5.173, 3.216] | .647 |
| Mouth | .007** | 4.649 [1.231, 8.068] | .008** | 0.006 [-3.634, 3.645] | .998 |
| Aud | .047* | 5.744 [1.045, 10.442] | .017* | 4.068 [-0.435, 8.571] | .077† |
| DMN | .972 | -0.177 [-5.407, 5.054] | .947 | -0.574 [-5.610, 4.461] | .823 |
| FP | .974 | -0.161 [-5.136, 4.814] | .949 | 0.446 [-4.333, 5.226] | .855 |
| VAN | .203 | 2.332 [-2.063, 6.728] | .298 | -1.505 [-5.770, 2.760] | .489 |
| CO | .223 | 3.952 [-0.776, 8.681] | .101 | 3.320 [-1.469, 8.109] | .174 |
| DAN | .496 | 2.072 [-1.906, 6.049] | .307 | 0.049 [-4.235, 4.332] | .982 |
| Sal | .484 | 0.502 [-5.037, 6.040] | .859 | 2.873 [-2.170, 7.916] | .264 |
| VOCAB |  |  |  |  |  |
| All | .140 | 2.770 [-1.149, 6.690] | .116 | 3.879 [-0.059, 7.817] | .054† |
| Hand | .164 | 2.213 [-1.070, 5.496] | .067† | 3.100 [-0.211, 6.412] | .186 |
| Vis | .355 | 2.052 [-0.756, 4.859] | .152 | 1.312 [-1.929, 4.553] | .428 |
| Mouth | .156 | 2.482 [-0.185, 5.148] | .068† | 2.221 [-0.607, 5.048] | .124 |
| Aud | .087† | 3.774 [0.265, 7.282] | .035* | 2.981 [-0.446, 6.408] | .088† |
| DMN | .379 | 1,505. -2.417 5.427] | .452 | 2.716 [-1.115, 6.546] | .165 |
| FP | .089† | -0.279 [-3.951, 3.392] | .882 | 3.636 [0.010, 7.261] | .049* |
| VAN | .134 | 2.708 [-0.597, 6.013] | .108 | 3.053 [-0.139, 6.245] | .061† |
| CO | .054† | 3.006 [-0.573, 6.585] | .100† | 4.402 [0.774, 8.031] | .017* |
| DAN | .075† | 2.839 [-0.175, 5.853] | .065† | 3.524 [0.236, 6.812] | .036* |
| Sal | .394 | 0.753 [03.432, 4.938] | .724 | 2.613 [-1.285, 6.510] | .189 |
| SPEED |  |  |  |  |  |
| All | .303 | 0.687 [-4.115, 5.490] | .779 | -2.934 [07.888, 2.021 | .246 |
| Hand | .151 | 0.857 [-3.168, 4.882] | .676 | -3.067 [-7.169, 1.035] | .143 |
| Vis | .264 | -1.411 [-4.853, 2.031] | .422 | -3.385 [-7.457, 0.686] | .103 |
| Mouth | .742 | 1.207 [-2.074, 4.487] | .471 | 0.292 [-3.261, 3.846] | .872 |
| Aud | .294 | -0.251 [-4.523, 4.020] | .908 | -2.951 [-7.160, 1.257] | .169 |
| DMN | .116 | 3.288 [-1.468, 8.045] | .175 | -1.263 [-6.137, 3.610] | .611 |
| FP | .295 | 1.278 [-3.373, 5.929] | .590 | -2.746 [-7.372, 1.880] | .245 |
| VAN | .784 | 0.630 [-3.445, 4.705] | .762 | -0.789 [-4.881, 3.303] | .706 |
| CO | .719 | -0.521 [-4.917, 3.874] | .816 | -1.814 [-6.362, 2.733] | .434 |
| DAN | .661 | 0.057 [-3.675, 3.790] | .976 | -1.609 [-5.719, 2.501] | .443 |
| Sal | .544 | 1.407 [-3.721, 6.535] | .591 | -1.363 [-6.187, 3.462] | .580 |

†*p*<.10; **p*<.05; ***p*<.01

B= unstandardized regression coefficient, with 95% Wald confidence intervals [LL: limit, UL: upper limit].

Abbreviations: All= overall internetwork rsFC; Vis= Visual; Aud= Auditory; DMN= default mode network; FP= fronto-parietal; VAN= ventral attention network; CO= cingulo-opercular; DAN= dorsal attention network; Sal= salience; FLUID= fluid reasoning; MEMORY= episodic memory; VOCAB= vocabulary; SPEED= perceptual speed.

Supplementary Table S2. Associations between network-based rsFC and demographic and cognitive variables.

|  | Overall | Hand | Vis | Mouth | Aud | DMN | FP | VAN | CO | DAN | Sal |
| --- | --- | --- | --- | --- | --- | --- | --- | --- | --- | --- | --- |
| Age | -0.159* | -0.145* | -0.120 | -0.022 | -0.164* | -0.101 | -0.139† | -0.192** | -0.246*** | -0.161* | -0.207** |
| Education | -0.024 | -0.007 | -0.035 | -0.025 | -0.020 | -0.023 | -0.004 | -0.045 | -0.022 | -0.045 | 0.010 |
| NART IQ | -0.162* | -0.097 | -0.175* | -0.058 | -0.177* | -0.140† | -0.146* | -0.167* | -0.183* | -0.134† | -0.125† |
| Caloric Intake | 0.043 | 0.026 | 0.042 | -0.017 | 0.036 | -0.002 | 0.092 | 0.041 | 0.070 | 0.063 | 0.046 |
| SPEED | 0.003 | -0.041 | 0.003 | 0.018 | -0.001 | 0.059 | 0.007 | -0.020 | -0.018 | -0.025 | -0.023 |
| FLUID | -0.134† | -0.106 | -0.130† | -0.127† | -0.038 | -0.160* | -0.136† | -0.044 | -0.044 | -0.091 | -0.124† |
| MEMORY | -0.033 | 0.020 | -0.079 | 0.016 | -0.004 | -0.076 | -0.061 | -0.011 | 0.049 | 0.043 | -0.014 |
| VOCAB | -0.212** | -0.161* | -0.251** | -0.095 | -0.188* | -0.145* | -0.203** | -0.141† | -0.216** | -0.187* | -0.193** |
| Race | 3.042* | 1.821 | 2.046 | 1.352 | 2.749† | 2.633† | 1.943 | 4.631* | 3.735* | 1.706 | 2.071 |
| Gender | 0.047 | 0.716 | 0.664 | 2.426 | 0.177 | 0.152 | 0.067 | 0.049 | 0.188 | 0.022 | 0.875 |

Values reflect Pearson's correlations (r) for continuous variables, and F-statistics from one-way analysis of variance for categorical variables.

†*p*<.10; **p*<.05; ***p*<.01; ****p*<.001.

Abbreviations: FLUID= fluid reasoning; MEMORY= episodic memory; VOCAB= vocabulary; SPEED= perceptual speed.

Supplementary Table S3. Scanning parameters for MRI sequences.

| Sequence | Parameters |
| --- | --- |
| BOLD | TE/TR: 20/2000 ms; Flip angle: 72°; In-plane resolution: 112×112; Slice thickness/gap: 3/0 mm; Slices: 37 |
| MPRAGE | TE/TR: 3/6.5 ms; Field of view: 256 mm; Flip angle: 8°; In-plane resolution: 256x256; Slice thickness/gap: 1/0 mm; Slices: 180 |
| dMRI | 55 directions; b: 800 s/mm2; TE/TR: 69/11032 ms; Flip angle: 90°; In-plane resolution 112x112; Voxels: 2x2x2 mm; Slices: 75 |
| FLAIR | TE/TR: 2800/11000 ms; Field of view: 23.0x17.96 cm; In-plane resolution: 256x189; Slice thickness/gap: 4/0.5 mm; Slices: 30 |

Abbreviations: BOLD= blood oxygen level-dependent; MPRAGE= magnetization-prepared rapid gradient-echo; dMRI= diffusion MRI; FLAIR= fluid-attenuated inversion recovery; TE= echo time; TR= repetition time.

Supplementary Figure S1. Regions of interest comprising each of the resting-state networks.


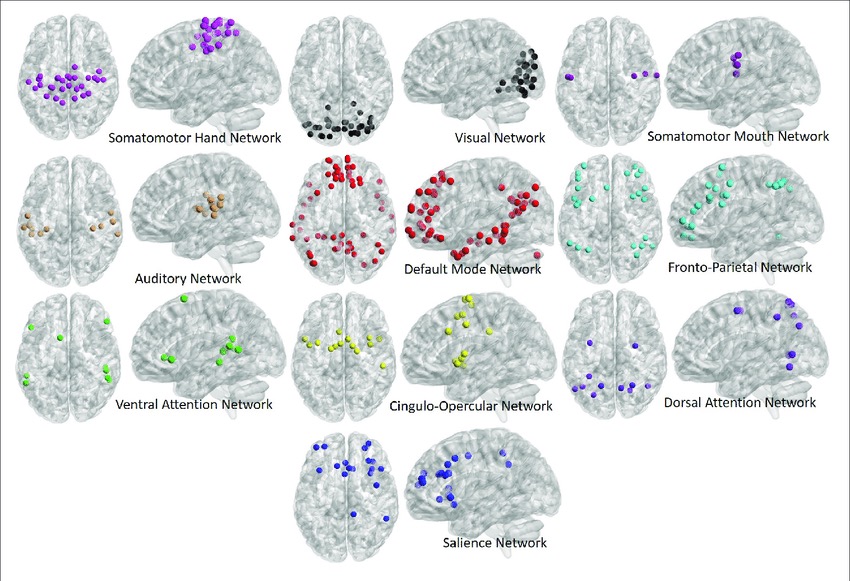


Regions of interest (ROIs) for each of the resting-state networks used in the current analyses, derived from Power et al. (2011) network parcellations. Figure reproduced with permission from Varangis et al. (2019).
